# The taxonomic revision of Asian *Aristolochia* (Aristolochiaceae) V: two new species from Yunnan, China

**DOI:** 10.3897/phytokeys.130.33933

**Published:** 2019-08-29

**Authors:** Xinxin Zhu, Xiaoqin Li, Shuai Liao, Guodong Li, Jinshuang Ma

**Affiliations:** 1 College of Life Sciences, Xinyang Normal University, Xinyang, Henan, 464000, China Shanghai Chenshan Plant Science Research Center, Chinese Academy of Sciences Shanghai China; 2 Shanghai Chenshan Plant Science Research Center, Chinese Academy of Sciences, Shanghai Chenshan Botanical Garden, Shanghai 201602, China Xinyang Normal University Xinyang China; 3 School of Life Sciences, East China Normal University, Shanghai 200241, China Shanghai Center for Plant Stress Biology, Chinese Academy of Sciences Shanghai China; 4 Faculty of Traditional Chinese Pharmacy, Yunnan University of Traditional Chinese Medicine, Kunming 650500, China University of Chinese Academy of Sciences Beijing China; 5 Shanghai Center for Plant Stress Biology, Chinese Academy of Sciences, Shanghai 201602, China East China Normal University Shanghai China; 6 University of Chinese Academy of Sciences, Beijing 100049, China Yunnan University of Traditional Chinese Medicine Kunming China

**Keywords:** *
Aristolochia
*, Baoshan, field expedition, morphology, taxonomy

## Abstract

*Aristolochia pseudoutriformis* X.X.Zhu & J.S.Ma, **sp. nov.** and *A. yangii* X.X.Zhu & J.S.Ma, **sp. nov.**, two new species from Yunnan, China, are described and illustrated here. The former is morphologically similar to *A. utriformis* and *A. forrestiana* and the latter is similar to *A. cucurbitoides* and *A. forrestiana*. According to Ma’s (1989a) classification, both new species belong to Aristolochia subgenus Siphisia on the basis of the 3-lobed gynostemium and oblong anthers that are adnate in pairs, opposite the gynostemium lobes. Meanwhile, the two new species are assessed as Vulnerable (VU D2) according to IUCN Red List criteria.

## Introduction

*Aristolochia* L. consists of about 550 species (González 2012) and is the largest genus in Aristolochiaceae (Hwang et al. 2003). Most species are distributed in the tropics and subtropics (Ma 1989a; Wanke et al. 2006). Three subgenera: subgenus Aristolochia, subgenus Siphisia and subgenus Pararistolochia are recognised based on morphological and molecular data (Wanke et al. 2006). China has 69 species according to Zhu et al. (2018). A key to the subgenera and a useful key to Asian species of Aristolochia subgenus Siphisia are provided by Do et al. (2015a).

During five field expeditions to Hundred Flowers Ridge in Longyang District, Baoshan, western Yunnan, two unknown species of *Aristolochia* were collected. Careful studies of the genus were undertaken, particularly the floral characteristics of those species in the adjacent regions, as well as morphological comparisons of the two unknown species with their related species. Meanwhile, through extensive fieldwork, careful examination of numerous specimens and colour photos and consultation of related publications, we confirm that they are two new species of *Aristolochia* which are described and illustrated in this study.

## Taxonomy

### Aristolochia pseudoutriformis

Taxon classificationPlantaePiperalesAristolochiaceae

X.X.Zhu & J.S.Ma sp. nov.

F74F8E12BF045981ABB578FD849990A8

urn:lsid:ipni.org:names:77201390-1

**Figure 1.**
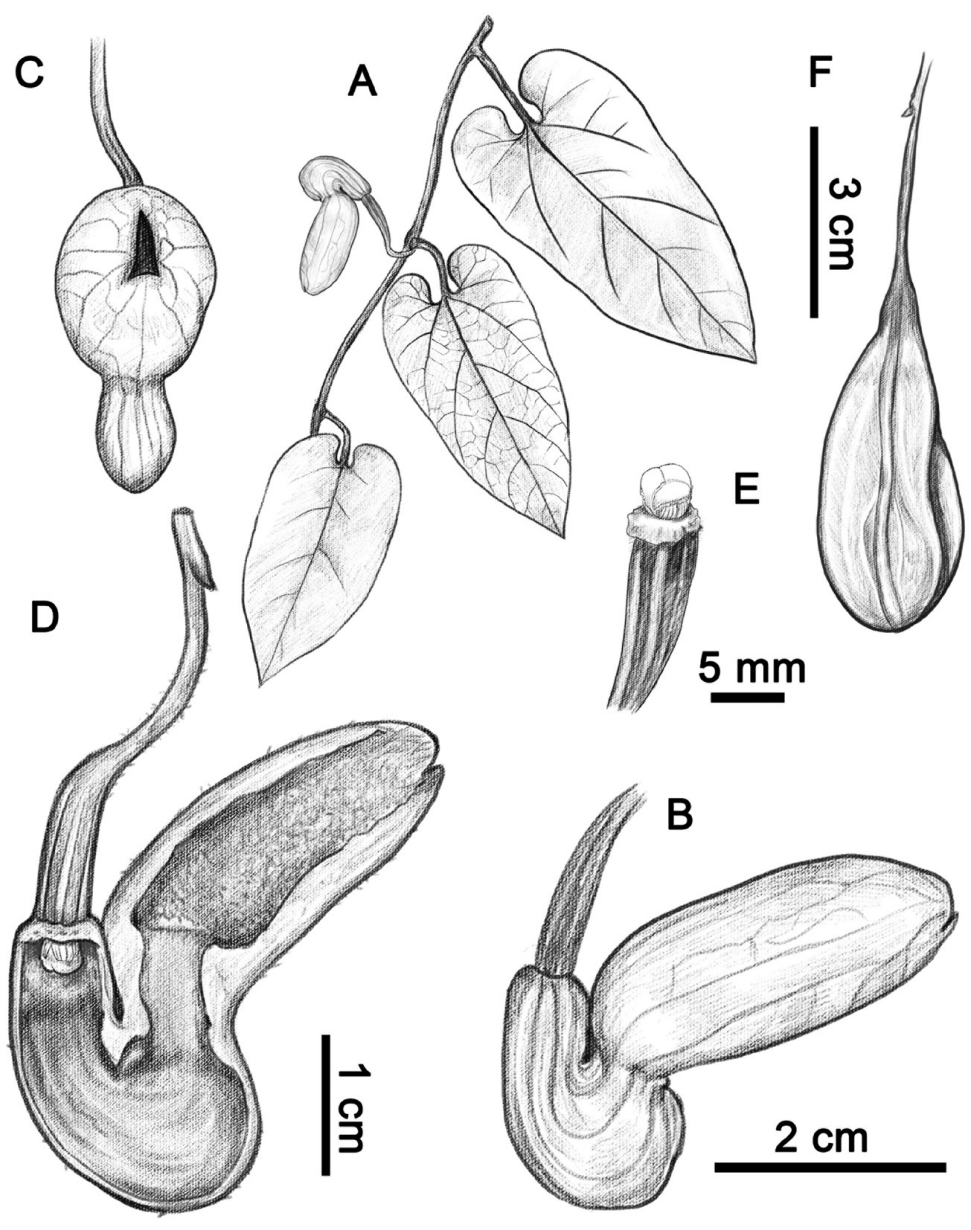
*Aristolochia pseudoutriformis* X.X.Zhu & J.S.Ma, sp. nov. **A** habit **B** flower (lateral view) **C** flower (front view) **D** opened flower (showing the inside structure) **E** anthers and gynostemium **F** fruit. Illustration by Shizhen Qiao.

**Figure 2.**
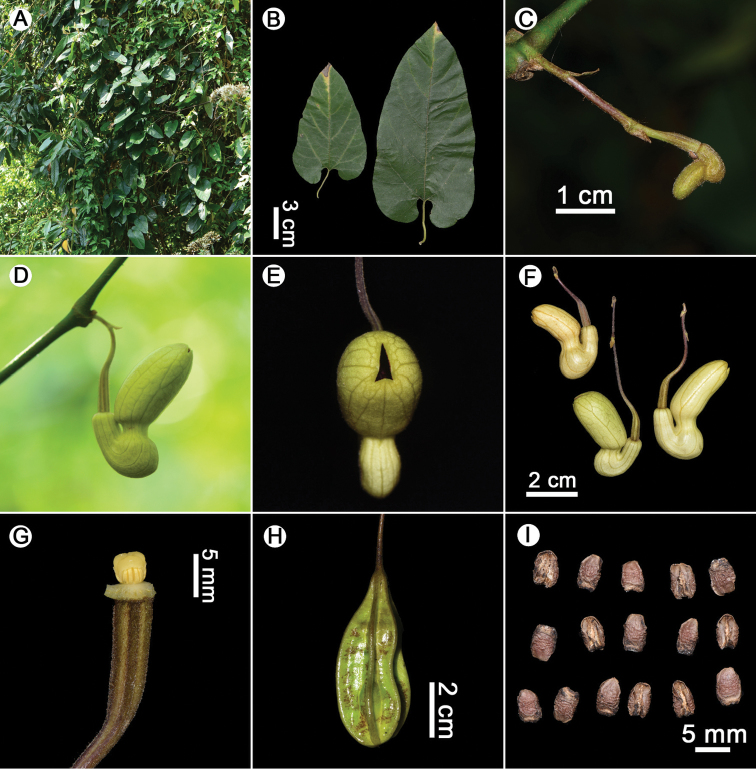
*Aristolochia pseudoutriformis* X.X.Zhu & J.S.Ma, sp. nov. **A** habit **B** leaves **C** flower bud **D** flower (lateral view) **E** flower (front view) **F** flowers **G** anthers and gynostemium **H** fruit **I** seeds. Photographed by Xinxin Zhu.

**Figure 3.**
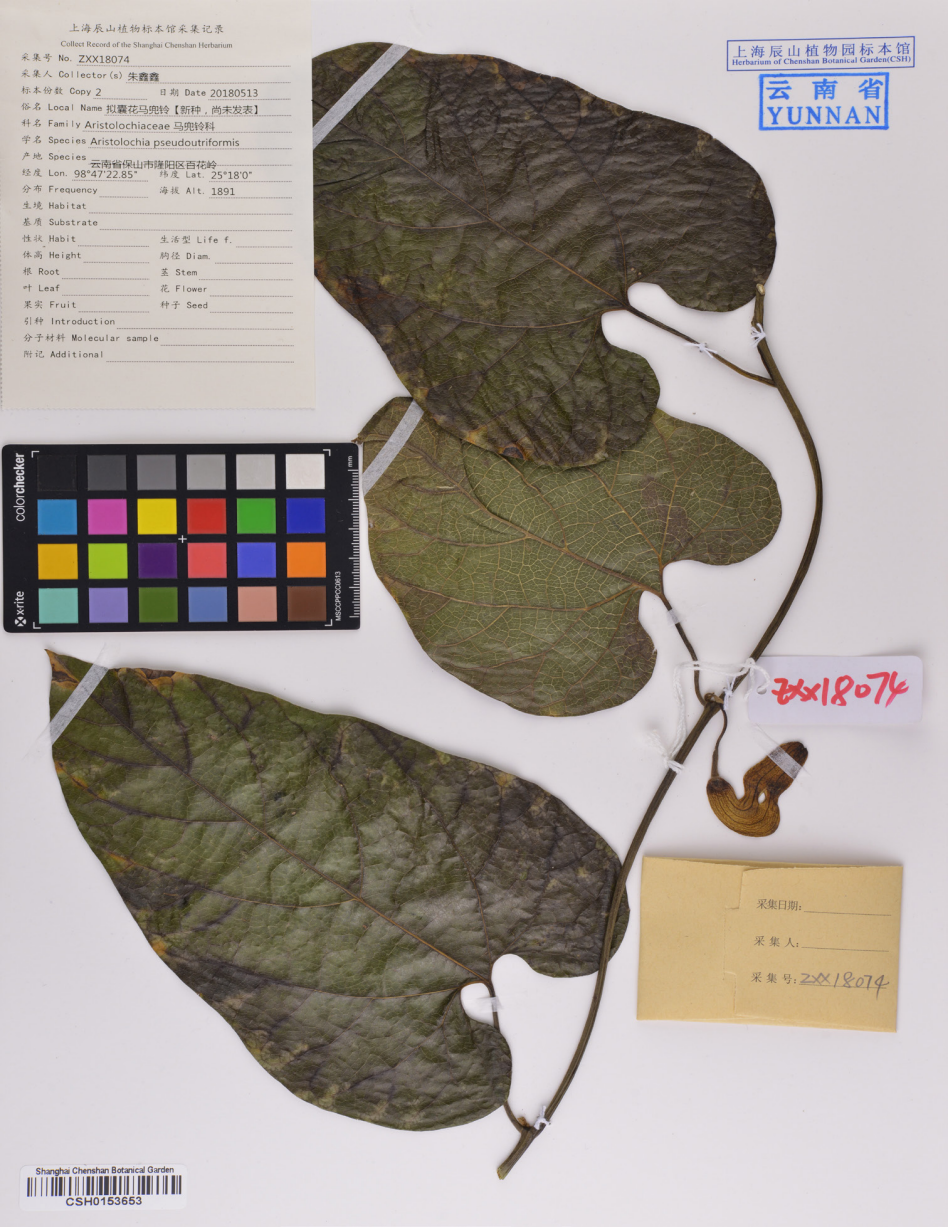
Holotype of *Aristolochia pseudoutriformis* X.X.Zhu & J.S.Ma, sp. nov. (CSH-0153653).

Figures 1 , 2 , 3 , 7A–C

#### Type.

CHINA. Yunnan: Baoshan, Longyang District, Hundred Flowers Ridge, 98°47.38'E, 25°18.00'N, 1891 m a.s.l., 13 May 2018, X.X.Zhu ZXX18074 (holotype: CSH [CSH-0153653!]; isotypes: CSH!, KUN!).

#### Diagnosis.

Similar to *Aristolochia utriformis* S.M.Hwang (Hwang 1981) and *A. forrestiana* J.S.Ma (Ma 1989b), but differs from the former in its lamina ovate to narrowly ovate (vs. ovate-lanceolate in *A. utriformis*), limb cylinder, forming obtuse angle with upper tuber, inside dark red, dense processes (vs. limb ovoid, straight extended from upper tube, inside black purple, sparse processes in *A. utriformis*) and throat ca. 6 mm in diam. (vs. ca. 1 mm in diam. in *A. utriformis*) and differs from the latter in its flower light yellow (vs. light brown or purple in *A. forrestiana*), limb slightly asymmetric, 2–3 × 1–1.7 cm; 3-lobed, lobes triangle or wide triangle; inside dark red (vs. asymmetric, 6–8 × 1.5–2 cm; 3-lobed, lobes lanceolate; inside black purple in *A. forrestiana*), as well as throat ca. 6 mm in diam. (vs. ca. 3mm in diam. in *A. forrestiana*). Detailed morphological comparisons are shown in Table 1 and Figure 7.

**Table 1.** Morphological comparisons amongst *Aristolochia pseudoutriformis*, *A. utriformis* and *A. forrestiana*.

**Characters**	***A. pseudoutriformis***	***A. utriformis***	***A. forrestiana***
Lamina	ovate to narrowly ovate	ovate-lanceolate	ovate to narrowly ovate
	10–22 × 7–13 cm	10–17 × 3–4 cm	7–21 × 3–10.5 cm
Calyx	light yellow	light yellow	light brown or purple
Limb	cylinder, slightly asymmetric, 2–3 cm long, forming obtuse angle with upper tuber, inside dark red, dense processes, 3-lobed, lobes triangle or wide triangle	ovoid, slightly asymmetric, 1–2 cm long, straight extended from upper tube, inside black purple, sparse processes, 3-lobed, lobes ovate-deltate	cylinder, asymmetric, 6–8 cm long, forming right angle with upper tuber, inside black purple, dense processes, 3-lobed, lobes lanceolate
Throat	ca. 6 mm in diam.	ca. 1 mm in diam.	ca. 3 mm in diam.

#### Description.

Semi-deciduous climbing shrubs. Stems terete. Petioles 2–5 cm long, densely pubescence; laminas ovate to narrowly ovate, 10–22 × 7–13 cm, adaxially sparsely pubescence, abaxially densely pubescence, base cordate, margin entire, apex acute; veins palmate, 2–3 pairs from base, lateral veins 3–5-paired. Flowers axillary, sometimes on stems, solitary or paired; pedicels 1.8–5 cm, densely brown villous; bractlets 1 or 2, ovate, 3–5 mm long, adaxially glabrous, abaxially densely villous. Calyx tube geniculately curved, light yellow, abaxially sparsely villous; basal tube 1.8–2.5 cm long, inside black purple at base, dark red above base, upper tube 1.2–1.8 cm long, inside dark red; limb saccate, cylinder, slightly asymmetric, 2–3 × 1–1.7 cm, forming obtuse angle with upper tuber, inside dark red, densely processes, 3-lobed, lobes triangle or wide triangle; throat ca. 6 mm in diam.. Anthers 6, oblong, ca. 1.5 mm long, adnate in 3 pairs to base of gynostemium, opposite to lobes. Gynostemium ca. 3 mm long, 3-lobed. Ovary terete, ca. 12 mm long, densely brown villous. Fruit stem ca. 4.5 cm long, sparsely puberulous. Capsule obovate-elliptic, distinctly 6-angled, sparsely puberulous on angles, ca. 6 × 2.5 cm (ca. 5 × 2.5 cm in dry specimens). Seeds ellipse, 5–6 × 3–4 mm, not winged, the adaxial surface deeply concave and the abaxial surface convex, wrinkled, both surfaces glabrous.

#### Phenology.

Flowering from March to May and fruiting from July to August.

#### Etymology.

The specific epithet refers to the similarity between the new species and *A. utriformis* in the morphology of flowers. The Chinese name is given as “拟囊花马兜铃”.

#### Distribution and habitat.

The new species is currently known to Longyang District, Baoshan, Yunnan, China. It grows in forests at an elevation of between 1890 m and 2260 m, together with *Castanopsis* ssp. (Fagaceae), *Disporum* sp. (Colchicaceae), *Elytranthe albida* (Bl.) Bl. (Loranthaceae), *Nervilia tahanshanensis* T.P.Lin & W.M.Lin (Orchidaceae), *Rubus* sp. (Rosaceae), etc.

#### IUCN Red List Category.

*Aristolochia pseudoutriformis* is known from only two populations, with fewer than five individuals at each site. Therefore, the new species is assigned a preliminary status of Vulnerable (VU D2) according to IUCN Red List Criteria (IUCN 2012), indicating a population with a very restricted area of occupancy (typically less than 20 km^2^) or number of locations (typically five or fewer).

#### Specimens Examined.

**CHINA. Yunnan**: Baoshan, Longyang District, Hundred Flowers Ridge, 30 Mar 2015, X.X.Zhu & Z.X.Hua ZH026 (CSH); 20 Apr 2017, X.X.Zhu ZXX17050 (CSH); 11 Aug 2018, X.X.Zhu & J.Wang ZXX18241 (CSH, KUN).

### Aristolochia yangii

Taxon classificationPlantaePiperalesAristolochiaceae

X.X.Zhu & J.S.Ma sp. nov.

D421836F38465EB1B7B7AC40F5F63300

urn:lsid:ipni.org:names:77201391-1

**Figure 4.**
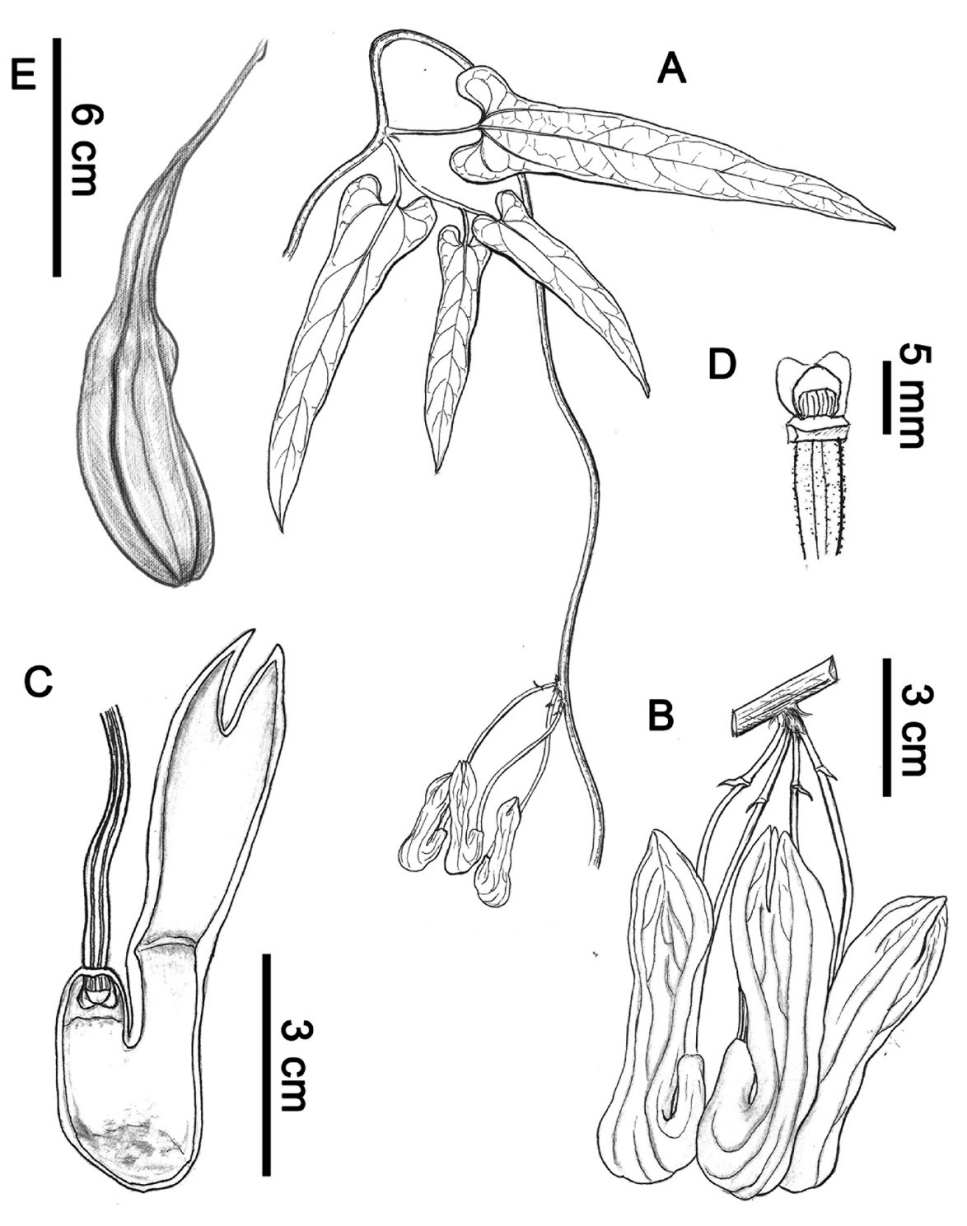
*Aristolochia yangii* X.X.Zhu & J.S.Ma, sp. nov. **A** habit **B** inflorescence **C** opened flower (showing the inside structure) **D** anthers and gynostemium **E** fruit. Illustration by Manhua Lin (**A**–**D**); Illustration by Shizhen Qiao (**E**).

**Figure 5.**
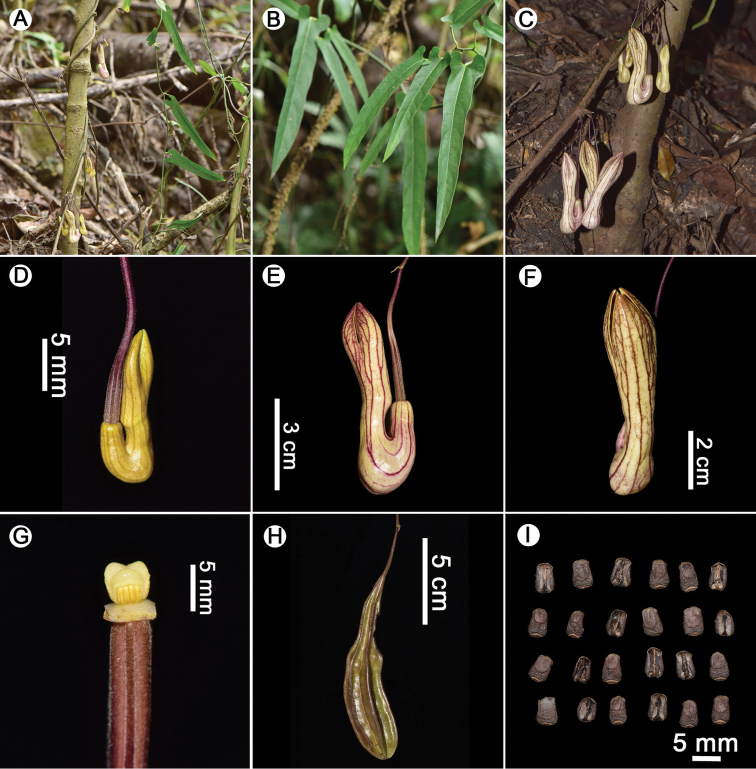
*Aristolochia yangii* X.X.Zhu & J.S.Ma, sp. nov. **A** habit **B** leaves **C** inflorescence **D** flower bud **E** flower (lateral view) **F** flower (front view) **G** anthers and gynostemium **H** fruit **I** seeds. Photographed by Xinxin Zhu.

**Figure 6.**
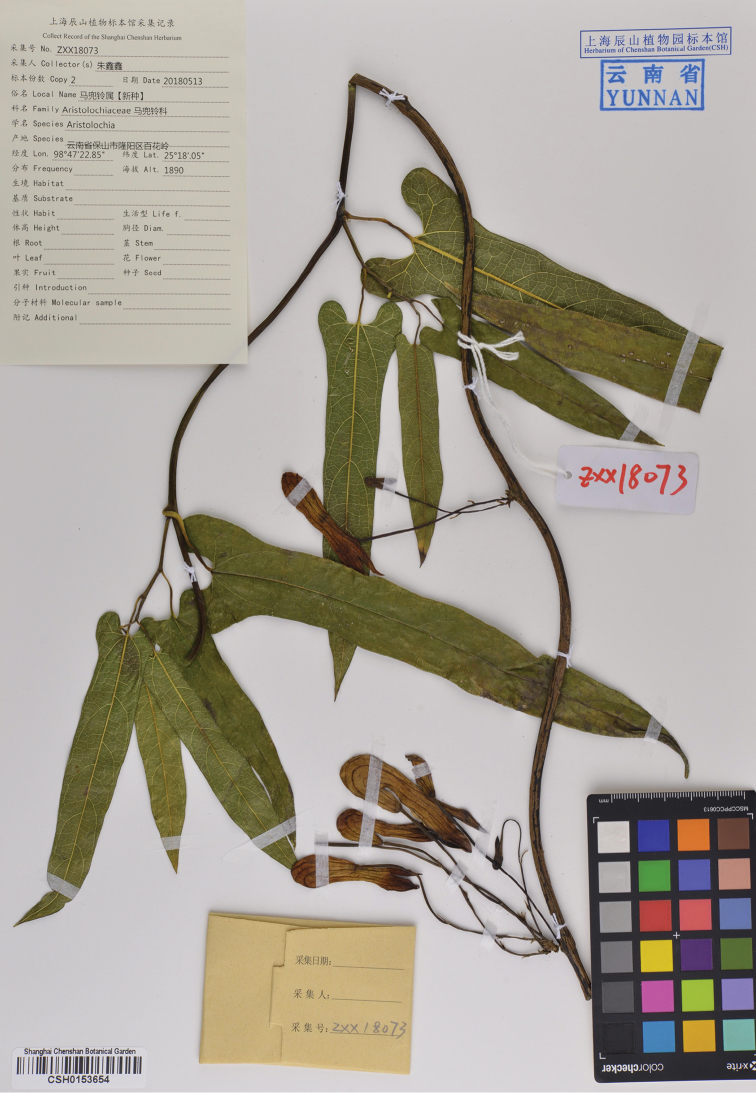
Holotype of *Aristolochia yangii* X.X.Zhu & J.S.Ma, sp. nov. (CSH-0153654).

**Figure 7.**
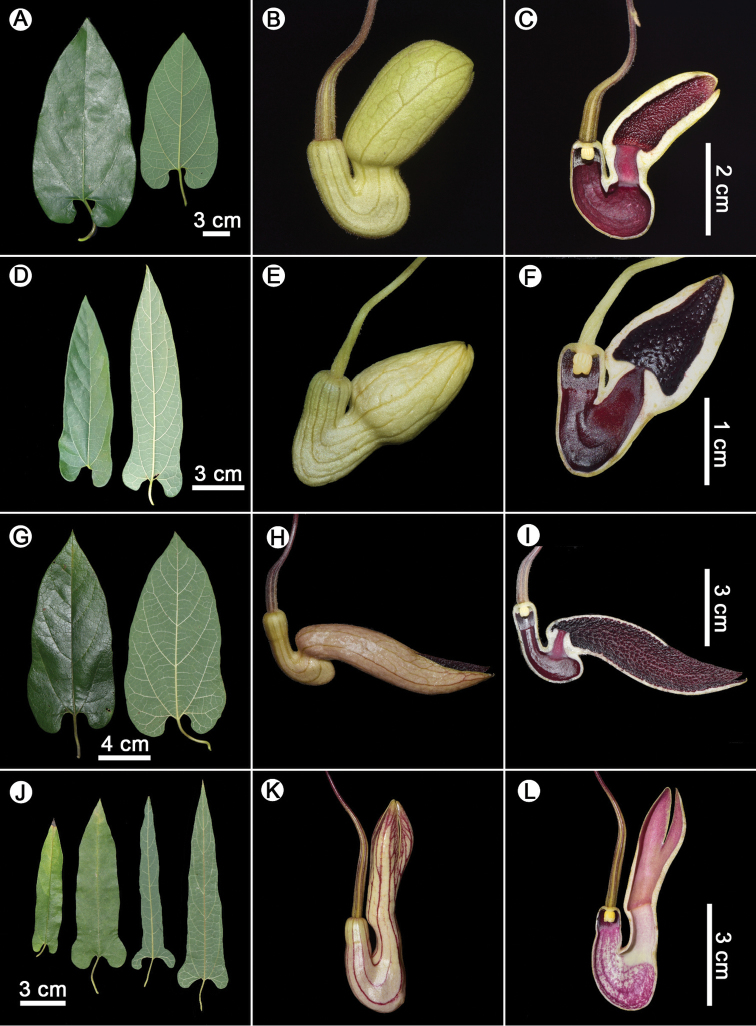
**A–C***Aristolochia pseudoutriformis* X.X.Zhu & J.S.Ma, sp. nov. **A** leaves **B** flower (lateral view) **C** opened flower (showing the inside structure). **D–F***A. utriformis* S.M.Hwang **D** leaves **E** flower (lateral view) **F** opened flower (showing the inside structure). **G–I***A. forrestiana* J.S.Ma **G** leaves **H** flower (lateral view) **I** opened flower (showing the inside structure). **J–L***A. yangii* X.X.Zhu & J.S.Ma. **J** leaves **K** flower (lateral view) **L** Opened flower (showing the inside structure). Photographed by Xinxin Zhu (**A–C**, **G–L**); Photographed by Lei Cai (**D–F**).

Figures 4 , 5 , 6 , 7J–L

#### Type.

CHINA. Yunnan: Baoshan, Longyang District, Hundred Flowers Ridge, 98°47.38'E, 25°18.00'N, 1890 m a.s.l., 13 May 2018, X.X.Zhu ZXX18073 (holotype: CSH [CSH-0153654!]; isotypes: CSH!, KUN!).

#### Diagnosis.

Similar to *Aristolochia cucurbitoides* C.F.Liang (Liang 1975) and *A. forrestiana* J.S.Ma (Ma 1989b), but differs from the former in its flower larger, basal tuber 2.5–3.5 cm long; limb 3.3–4.7 cm long; deeply 3-lobed, lobes ovate-deltoid, 1.6–2.4 cm long (vs. flower smaller, basal tuber ca. 2 cm long; limb ca. 2 cm long; shallowly 3-lobed, lobes lanceolate-acuminate, 0.5–0.7 cm long in *A. cucurbitoides*) and differs from the latter in its lamina lanceolate to hastate, 8–24.5 × 1.2–5.5 cm, base auriculate to cordate (vs. lamina ovate to narrowly ovate, 7–21 × 3–10.5 cm, base cordate in *A. forrestiana*), calyx yellowish-white (vs. light brown or purple in *A. forrestiana*), as well as limb symmetric, 3.3–4.7 cm long, straight extended from upper tube and parallel to it; deeply 3-lobed, lobes ovate-deltoid; inside pinkish or ochre without processes (vs. asymmetric, 6–8 cm long, forming right angle with upper tuber; 3-lobed, lobes lanceolate; inside black purple with dense processes in *A. forrestiana*). Detailed morphological comparisons amongst the three species are summarised in Table 2 and comparisons between *A. pseudoutriformis* and *A. forrestiana* are also shown in Fig. 7.

#### Description.

Semi-deciduous climbing shrubs. Stems terete. Petioles 1–4 cm long, densely pubescence; laminas lanceolate to hastate, 8–24.5 × 1.2–5.5 cm, adaxially glabrous or sparsely pubescence along medial vein, abaxially densely pubescence, base auriculate to cordate, margin entire, apex acute; veins palmate, 2–3 pairs from base, lateral veins 6–15-paired. Cymes on old woody stems or axillary, 1–2-flowered, in clusters of 1 to numerous; pedicels 5–7 cm long, sparsely pubescence; bractlets 1 or 2, ovate-lanceolate, 3–5 mm long, adaxially glabrous, abaxially densely brown villous. Calyx tube geniculately curved, yellowish-white with distinct purple stripe, abaxially subglabrous or sparsely villous; basal tuber 2.5–3.5 cm long, inside black purple at base, white mix with more or less reddish-violet above base, upper tube 2.5–3.5 cm long, inside white mix with reddish-violet, limb cylindric, symmetric, 3.3–4.7 cm long, straight extended from upper tube and parallel to it, inside pinkish or ochre, deeply 3-lobed, lobes ovate-deltoid, 1.6–2.4 cm long; throat ca. 7 mm in diam. Anthers 6, oblong, ca. 2 mm long, adnate in 3 pairs to base of gynostemium, opposite to lobes. Gynostemium ca. 3.5 mm long, 3-lobed. Ovary terete, 15–25 mm long, densely brown villous. Fruit stem purple red, ca. 6.5 cm long, sparsely puberulous. Capsule narrowly obovate-elliptic, distinctly 6-angled, nearly glabrous, ca. 10.5 × 2.5 cm (ca. 8.5 × 2 cm in dry specimens). Seeds ovate-elliptic, 5–5.5 × 3.5–4 mm, not winged, the adaxial surface deeply concave and the abaxial surface convex, wrinkled, both surfaces glabrous.

#### Phenology.

Flowering May and fruiting from July to August.

#### Etymology.

The new species is named after Zhiguang Yang, who first discovered this rare species and who accompanied us on a number of subsequent field expeditions in Hundred Flowers Ridge, Baoshan, Yunnan. The Chinese name is given as “杨氏马兜铃”.

#### Distribution and habitat.

The new species is currently known to Longyang District, Baoshan, Yunnan, China. It grows in forests at an elevation of between 1880 m and 2130 m, together with *Castanopsis* ssp. (Fagaceae), *Disporum* sp. (Colchicaceae), *Elytranthe albida* (Bl.) Bl. (Loranthaceae), *Nervilia tahanshanensis* T.P.Lin & W.M.Lin (Orchidaceae), *Rubus* sp. (Rosaceae), etc.

#### IUCN Red List Category.

*Aristolochia yangii* is known from only three populations, with fewer than ten individuals seen at each site. Therefore, the new species is assigned a preliminary status of Vulnerable (VU D2) according to IUCN Red List Criteria (IUCN 2012), indicating a population with a very restricted area of occupancy (typically less than 20 km^2^) or number of locations (typically five or fewer).

#### Specimens Examined.

**CHINA. Yunnan**: Longyang District, 30 Mar 2015, X.X.Zhu & Z.X.Hua ZH028 (CSH); 4 June2017, X.X.Zhu ZXX17074 (CSH); 11 Aug 2018, X.X.Zhu & J.Wang ZXX18242 (CSH).

## Discussion

*Aristolochia pseudoutriformis* is morphologically similar to *A. utriformis* (Figs 7D–F) in the shape and colour of flower, but they can be distinguished by the morphology of lamina and limb and the size of throat. It is also similar to *A. forrestiana* (Figs 7G–I) in the morphology of lamina, whereas they differ in the shape and colour of flower, the morphology of limb, as well as the size of throat (see Table 1 and Fig. 7)

*Aristolochia yangii* is similar to *A. cucurbitoides* in the shape of lamina, but they can be distinguished by the morphology of flower. It is also similar to *A. forrestiana* (Figs 7G–I) in the size of flower, whereas they differ in the shape and colour of flower, the morphology of limb, as well as the shape of lamina (summarised in Table 2 and comparison of *A. pseudoutriformis* and *A. forrestiana* is also shown in Fig. 7).

**Table 2.** Morphological comparisons amongst *Aristolochia yangii*, *A. cucurbitoides* and *A. forrestiana*.

**Characters**	***A. yangii***	***A. cucurbitoides***	***A. forrestiana***
Lamina	lanceolate to hastate, 8–24.5 × 1.2–5.5 cm, base auriculate to cordate	trullate-lanceolate, ovate-lanceolate, or lanceolate, 12–22 × 2.5–4.5 cm	ovate to narrowly ovate, 7–21 × 3–10.5 cm
Calyx	Yellowish-white; inside of basal tuber black purple at base, white mix with more or less reddish-violet above base, inside of upper tube white mix with reddish-violet	undocumented	light brown or purple; inside of tuber black purple
Limb	3.3–4.7 cm long symmetric, straight extended from upper tube and parallel to it, inside pinkish or ochre, smooth, deeply 3-lobed, lobes ovate-deltoid, 1.6–2.4 cm long	ca. 2 cm long, slightly asymmetric, straight extended from upper tube, inside undocumented, shallowly 3-lobed, lobes lanceolate-acuminate, 0.5–0.7 cm long	6–8 cm long, asymmetric, forming right angle with upper tuber, inside black purple with dense processes, 3-lobed, lobes lanceolate, ca. 2.5 cm long

Both two new species with horseshoe-shaped perianth, the 3-lobed gynostemium and each lobe consisting of one pair oblong stamens belongs to Aristolochia subgenus Siphisia (Ma 1989a). These new discoveries, along with many new species recently described from China and neighbouring countries (Phuphathanaphong 2006; Liu and Deng 2009; Xu et al. 2011; Yao 2012; Huang et al. 2013, 2015; Wu et al. 2013; Do et al. 2014, 2015a, 2015b, 2015c, 2015d, 2017; Huong et al. 2014; Lu and Wang 2014; Ohi-Toma et al. 2014; Ravikumar et al. 2014; Zhu et al. 2015, 2016, 2017a, 2017b, 2018; Gong et al. 2018; Yang et al. 2018) provide evidence that the genus *Aristolochia* and especially Aristolochia subgenus Siphisia is very diverse in South-East Asia. We predict that more new species of *Aristolochia* will be found after extensive investigations in this region.

## Supplementary Material

XML Treatment for Aristolochia pseudoutriformis

XML Treatment for Aristolochia yangii
